# Spatial specificity and inheritance of adaptation in human visual cortex

**DOI:** 10.1152/jn.00167.2015

**Published:** 2015-06-10

**Authors:** Jonas Larsson, Sarah J. Harrison

**Affiliations:** Department of Psychology, Royal Holloway, University of London, Egham, United Kingdom

**Keywords:** adaptation, extrastriate visual areas, fMRI, V1, visual cortex

## Abstract

Adaptation at early stages of sensory processing can be propagated to downstream areas. Such inherited adaptation is a potential confound for functional magnetic resonance imaging (fMRI) techniques that use selectivity of adaptation to infer neuronal selectivity. However, the relative contributions of inherited and intrinsic adaptation at higher cortical stages, and the impact of inherited adaptation on downstream processing, remain unclear. Using fMRI, we investigated how adaptation to visual motion direction and orientation influences visually evoked responses in human V1 and extrastriate visual areas. To dissociate inherited from intrinsic adaptation, we quantified the spatial specificity of adaptation for each visual area as a measure of the receptive field sizes of the area where adaptation originated, predicting that adaptation originating in V1 should be more spatially specific than adaptation intrinsic to extrastriate visual cortex. In most extrastriate visual areas, the spatial specificity of adaptation did not differ from that in V1, suggesting that adaptation originated in V1. Only in one extrastriate area—MT—was the spatial specificity of direction-selective adaptation significantly broader than in V1, consistent with a combination of inherited V1 adaptation and intrinsic MT adaptation. Moreover, inherited adaptation effects could be both facilitatory and suppressive. These results suggest that adaptation at early visual processing stages can have widespread and profound effects on responses in extrastriate visual areas, placing important constraints on the use of fMRI adaptation techniques, while also demonstrating a general experimental strategy for systematically dissociating inherited from intrinsic adaptation by fMRI.

neural adaptation is a ubiquitous phenomenon that has been observed at multiple levels of the visual system, from the retina to extrastriate visual cortex (Kohn 2007; Solomon and Kohn 2014). Several differing explanations of the mechanisms and functional role of adaptation have been proposed, including neural fatigue (Barlow 1990; Carandini and Ferster 1997; Sanchez-Vives et al. 2000), gain control (Abbott et al. 1997; Ohzawa et al. 1982), efficient coding (Barlow and Foldiak 1989; Benucci et al. 2013; Gutnisky and Dragoi 2008; Muller et al. 1999; Webster 2011), or energetic efficiency (Benucci et al. 2013). Most research into adaptation mechanisms has focused on the properties of the neuronal populations undergoing adaptation, but there is growing recognition that adaptation effects can also be propagated to downstream neuronal populations (Dhruv and Carandini 2014; Patterson et al. 2014a, 2014b; Solomon et al. 2004). Such “cascaded” or “inherited” adaptation complicates interpretation of the mechanisms, or functional role, of adaptation in several ways. First, adaptation-induced changes in responses measured in one population of neurons may be partly or wholly inherited from an upstream area rather than being intrinsic to the neurons under study (Kohn and Movshon 2003). Second, neural adaptation at early processing stages can potentially derail downstream computations (Kohn and Movshon 2004; Patterson et al. 2014a; Tolias et al. 2005; Xu et al. 2008), especially if later stages are unable to adjust to adaptation-induced changes of their inputs (Dhruv and Carandini 2014). Third, adaptation can result in both decreases and increases in output spiking activity (Camp et al. 2009; Webb et al. 2005; Wissig and Kohn 2012), making the effects of inherited adaptation potentially very complex.

Inherited adaptation poses a particular problem for functional neuroimaging studies that use adaptation to infer neuronal response properties of brain areas [“functional magnetic resonance imaging (fMRI) adaptation”] (Krekelberg et al. 2006). If adaptation effects are dominated by inherited adaptation, the selectivity of adaptation may not reflect the response properties of the area under measurement but rather the properties of the upstream area where adaptation originated. Inferences about neuronal response properties obtained by fMRI adaptation can thus be misleading, unless the relative contributions of inherited and intrinsic adaptation can be quantified. Two seminal studies of motion adaptation in macaque MT (Kohn and Movshon 2003; Priebe et al. 2002) demonstrated that these two contributions can be dissociated by measuring the spatial extent of adaptation as a measure of receptive field (RF) sizes of the neurons undergoing adaptation, revealing that the bulk of motion adaptation in this area is inherited from V1. However, no comparable experiment has been carried out to dissociate intrinsic and inherited contributions to fMRI adaptation.

In this study we have applied a variation of the methods used by Kohn and Movshon (2003) and Priebe et al. (2002) to dissociate intrinsic and inherited fMRI adaptation effects for two basic visual stimulus features, motion and orientation. Using fMRI, we measured the spatial specificity of orientation- and direction-selective adaptation by systematically varying the spatial offset between adapting and test stimuli and quantifying the strength of adaptation at each offset. Since the spatial specificity of adaptation should reflect the extent of RFs where adaptation originated, this provided a way of distinguishing adaptation inherited from V1 from adaptation intrinsic to higher visual areas. Specifically, we predicted that adaptation originating in V1 should show tighter spatial specificity than adaptation that originated in higher extrastriate areas, as a result of the relatively smaller RFs of the neural population in V1. By comparing the spatial specificity of adaptation in extrastriate visual areas with that in V1, we were able to determine the relative contribution of inherited versus intrinsic adaptation to address the following three questions that have particular significance for the interpretation of fMRI adaptation effects.

First, given that many stimulus features can selectively drive neurons at multiple stages of processing, does adaptation to these features also occur at each processing stage, or only once (at the first stage)? For example, neurons selective for motion direction are found both in V1 and MT, implying that adaptation to motion could potentially occur in both areas. Previous research has found evidence of both inherited adaptation (Kohn and Movshon 2003) and intrinsic adaptation (Priebe et al. 2002) in MT, but differences in timescales and stimuli between these studies make it difficult to draw general conclusions from the results. For other stimulus features, such as orientation, it is not known whether adaptation can also take place at multiple visual processing stages or only at the first level. Several fMRI adaptation studies have found orientation-selective adaptation in multiple visual areas (e.g., Larsson et al. 2006, 2010; Montaser-Kouhsari et al. 2007), which could reflect multiple stages of adaptation or inherited adaptation effects or both.

Second, is inherited adaptation selective, i.e., does adaptation to a particular stimulus feature propagate preferentially to areas selective for the adapted stimulus feature or equally to all downstream areas? For example, are the effects of direction-selective adaptation in V1 preferentially inherited by motion-responsive extrastriate visual areas such as MT or V3A (Tootell et al. 1995, 1997) or equally by all extrastriate areas receiving V1 input? The degree to which inherited adaptation is selective is critical for the interpretation of fMRI adaptation effects. If adaptation is inherited primarily by areas selective for the adapted stimulus feature, the selectivity of adaptation would be expected to reflect neuronal response selectivity even in the presence of inherited adaptation. Conversely, if inherited adaptation is nonselective, this relationship would not hold, confounding the interpretation of fMRI adaptation as a measure of response selectivity.

Third, it is not clear whether adaptation only changes the responsiveness of neurons to an adapting stimulus or can also influence the effectiveness of adapting neurons at driving downstream stages. For example, does adaptation in V1 modulate how strongly V1 outputs drive responses in extrastriate visual areas? Recent evidence suggests that the coupling between LGN and V1 is unaffected by subcortical adaptation (Dhruv and Carandini 2014), but it is not known whether this is true also for intracortical connections. Moreover, some evidence indicates that adaptation may change the coupling between oxygen metabolism and blood oxygenation [blood oxygenation level dependent (BOLD)] signals measured by fMRI (Moradi and Buxton 2013), which could potentially lead to changes in interareal coupling of the fMRI signals even in the absence of changes in neural coupling. Such changes could potentially further complicate interpretation of fMRI adaptation studies, above and beyond those associated with inherited adaptation per se.

Our results suggest that adaptation to both stimulus orientation and motion direction originates largely in V1, with little evidence for additional adaptation at higher stages. The only exception was in MT, where a proportion of direction-selective adaptation was likely intrinsic to this area, but only for broadband motion stimuli. For both stimulus features, adaptation in V1 was propagated to a majority of extrastriate visual areas, regardless of the stimulus selectivity of these areas. Moreover, adaptation did not change the coupling between V1 and extrastriate visual areas, implying that downstream areas do not adjust to adaptation-induced changes in input drive.

## MATERIALS AND METHODS

### Subjects

Eleven subjects aged between 18 and 39 yr took part in the experiment. Subjects were naive to the purpose of the experiment, with the exception of one author who took part in testing. Ethical approval was obtained from the research ethics committee of the Department of Psychology at Royal Holloway, University of London. Subjects gave informed written consent to participate, and the experiments were undertaken in compliance with safety guidelines for magnetic resonance imaging (Kanal et al. 2002).

### Experimental Design

**Fig. 1.**
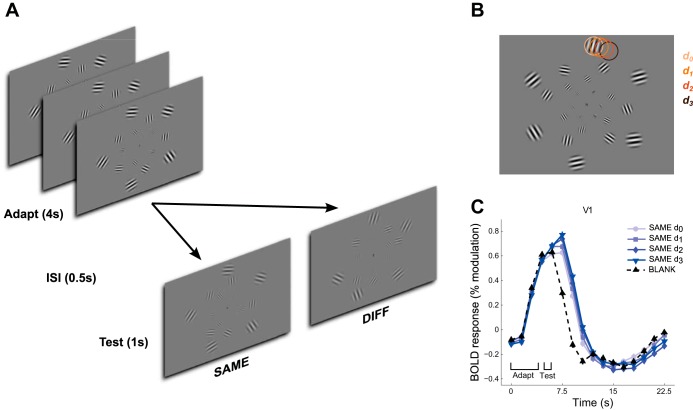
Stimuli and experimental design. *A*: experimental design. On each trial, the adapter stimulus was shown for 4 s, followed after a 500-ms interstimulus interval (ISI) by the probe stimulus shown for 1 s. Probe stimuli could have the same direction [broadband motion (BM), narrowband motion (NM) conditions] or orientation [static orientation (SO) condition] as the adapter (SAME trials) or the opposite direction/orthogonal orientation (DIFF trials). In BLANK trials, only adapter stimuli were shown. *B*: example of grating stimulus (conditions NM and SO). In each offset condition *d*_0_–*d*_3_, probe stimulus patches were displaced in random directions in the polar dimension from the adapter patch locations by 1 of 4 offsets (*d*_0_–*d*_3_), with *d*_0_ corresponding to zero displacement and *d*_3_ to 0.825 patch width (indicated schematically by colored circles at *top*). *C*: BOLD fMRI responses to BM stimuli in V1. Time courses correspond to the event-related responses (averaged across subjects) to SAME trials for each adapter-probe offset and for BLANK trials (adapter only).

We measured the postadaptation fMRI responses to arrays of stimulus patches, with probe patches spatially offset relative to adapter patches according to four offset conditions (corresponding to increasing displacement between adapter and probe patches) (Fig. 1, *A* and *B*). Stimulus patterns were displayed within each patch according to one of three stimulus conditions: flickering sinusoidal gratings (static orientation, SO), moving sinusoidal gratings (narrowband motion, NM), and moving random dot patterns (broadband motion, BM).

Each subject took part in three main (adaptation) experiments, corresponding to the three stimulus types, that were run in separate scanning sessions. The experiments differed in the details of the stimuli displayed within patches but were otherwise identical. An event-related experimental design was used. On each trial, we measured the visually evoked fMRI response to brief (1 s) presentations of a probe stimulus following a 4-s adapter stimulus and a 0.5-s interstimulus interval. There were three trial types: SAME, DIFF, and BLANK. On SAME trials, the probe stimuli moved in the same direction (NM/BM) or had the same grating orientation (SO) as the adapter stimuli (Fig. 1*A*). On DIFF trials, the probe stimuli moved in the opposite direction (NM/BM) or had the orthogonal orientation (SO). On BLANK trials, only the adapter stimulus was shown, followed by a blank screen. Probe patches were shown at four different spatial offsets from adapter patches, yielding nine different stimulus conditions (4 each of SAME and DIFF, plus BLANK). After the probe presentation, the screen remained blank (except for the fixation stimulus described below) for an interval that was randomly allocated to be 0.5, 2, or 3.5 s, resulting in total trial durations of 6, 7.5, or 9 s. Trial onsets were aligned with the scanner image acquisition.

Each experimental run (scan) started with a 15-s continuous adaptation period. This was followed by 45 trials (5 repeats of each type) described above, with trial order randomly permuted within each block of 9 trials. At the end of each scan, the screen remained blank for 12 s, resulting in a total scan time of 364.5 s. Each session consisted of 10 scans, yielding a total of 50 trials per trial type and subject.

Concurrent with the adaptation trials, subjects performed an attention-demanding rapid-serial-visual-presentation (RSVP) task at the center of fixation that was identical across scans and stimulus conditions. Subjects were required to count the number (0–3) of target letters “X” in a rapid (150 ms/letter) stream of distractor letters shown at the center of gaze and to respond by pressing one of four response keys. Each letter stream lasted 3, 4, or 5 s (randomly allocated) and was followed by a 1-s response window indicated by the presentation of a small black cross at fixation. Task feedback was provided by changing the color of the fixation cross to green after a correct response and to red after an incorrect response. RSVP trials were run back to back, with timing independent of and asynchronous with the experimental stimuli. We have previously shown that this task is effective at controlling and equating spatial attention across different trials and stimulus conditions in experiments using similar event-related designs (Larsson and Heeger 2006; Larsson and Smith 2012; Montaser-Kouhsari et al. 2007).

### Visual Stimuli

Stimuli were back-projected on a rear projection screen at a refresh rate of 60 Hz and viewed through a front-silvered mirror. Stimuli consisted of arrays of 24 circular patches (stimulus apertures) presented on a midgray background (Fig. 1*B*). The patches were arranged in four concentric rings around a central fixation cross and centered at eccentricities of 3.00°, 5.44°, 8.61°, and 12.50° visual angle. Patch size increased linearly with eccentricity from 1° in the innermost ring to 4.17° in the outermost ring, such that patches subtended the same fraction of the visual field in the polar dimension at all four eccentricities. Adapter stimuli were created by placing the six patches within each ring with even spacing (0.33 rad), and rotating each ring by half the interpatch polar spacing relative to the preceding ring, before the addition of random polar jitter to each polar location drawn from a rectangular distribution spanning −0.25 to 0.25 of the interpatch polar spacing (i.e., ±0.083 rad). Patch locations within adapter stimuli were randomized across scans but were held constant throughout each scan. For probe stimuli, patches were offset relative to patch locations in the adapter stimulus by 0, 0.042, 0.083, or 0.125 rad in the polar direction, corresponding to 0, 0.275, 0.55, and 0.825 patch widths, in offset *conditions 1–4*, respectively (Fig. 1*B*). The direction of offset was randomly clockwise or counterclockwise for each individual probe and trial.

#### Narrowband motion.

NM stimuli consisted of sinusoidal gratings drifting at a speed of 5 cycles/s, windowed by an envelope defined by a raised cosine contrast function with an exponent of 0.5. The number of cycles per patch was constant across eccentricities, meaning that the spatial frequency of the gratings varied inversely with eccentricity from 4 cycles/° for the innermost patches to 0.96 cycles/° for the outermost patches, corresponding to physical stimulus speeds of 1.25°/s to 5.25°/s, respectively. Adapter stimuli had a Michelson contrast of 1 [root mean square (RMS) contrast = 0.7], and probe stimuli had a contrast of 0.2 (RMS contrast = 0.14). Grating orientation was randomly allocated for each patch but held constant throughout a scan. Similarly, the direction of motion of each adapter patch was random but constant within a scan. For probe stimuli, grating orientation was identical to that of the corresponding adapter patch. The direction of motion of probe gratings was either identical (SAME condition) or opposite (DIFF condition) to the direction of the corresponding adapter stimulus gratings.

#### Broadband motion.

BM stimuli consisted of drifting random dot patterns windowed by a raised cosine contrast envelope as for the NM stimuli. Dot size was 0.067° visual angle for patches in the innermost ring, and all spatial parameters were linearly scaled with eccentricity for patches in subsequent rings. Dot density within each patch was 0.2; half of the dots within each patch were black, and half were white. Adapter and probe stimuli both had an RMS contrast of 0.45. Within each patch, dot motion was 100% coherent. Dots moved at a speed of 1.33°/s for patches in the innermost ring, increasing linearly with eccentricity to 5.54°/s for patches in the outermost ring. The direction of motion of dots within each adapter patch was randomly allocated but held constant throughout a scan. The direction of motion within each patch was either identical to (SAME condition) or opposite to (DIFF condition) the direction of motion within the corresponding adapter stimulus patches.

#### Static orientation.

SO stimuli were sinusoidal gratings identical to those used in the NM stimuli, but instead of drifting in a single direction the spatial phase of the gratings changed randomly between one of four phases (0°, 45°, 90°, and 135°) at a rate of 20 Hz. The orientation of each static grating patch in the adapter stimulus was randomly allocated but held constant throughout a scan. The orientation of probe stimulus gratings was either parallel (SAME condition) or orthogonal (DIFF condition) to their corresponding adapter stimulus gratings.

### MRI Acquisition

Visually evoked cortical BOLD fMRI responses were measured by T_2_*-weighted gradient-recalled echoplanar imaging on a 3-T whole-body MR scanner (Magnetom Trio; Siemens, Erlangen, Germany) equipped with a custom eight-channel posterior-head array coil (Stark Contrast; Erlangen, Germany). fMRI data were acquired from 19 oblique slices either roughly parallel to the calcarine sulcus and covering the occipital and temporal cortex (8 subjects) or at an approximately perpendicular angle that covered the occipital cortex and the thalamus (3 subjects). Scan parameters were identical regardless of slice positioning [voxel size 3 × 3 × 3 mm, time repetition (TR) = 1,500 ms, time echo (TE) = 34 ms, flip angle = 85°]. On each session, a whole-brain anatomical MR volume was acquired and used for spatial coregistration of data across sessions [voxel size 1 × 1 × 1 mm, MPRAGE sequence, TR = 1,830 ms, time to inversion (TI) = 1,100 ms, TE = 5.6 ms, flip angle = 11°]. In a separate session, a high-resolution high-contrast T_1_-weighted anatomical MR volume of each subject was acquired [voxel size 1 × 1 × 1 mm, MDEFT sequence (Deichmann 2006)] (TR = 7.9 ms, TI = 910 ms, TE = 2.5 ms, flip angle = 16°), which was used for coregistration of data across sessions and cortical surface reconstruction.

### MRI Preprocessing

Functional image volumes acquired at different time points within a scanning session were spatially aligned with the motion-correction software mcflirt (Jenkinson et al. 2002), linearly detrended, and high-pass filtered with a cutoff of 0.022 Hz. Data from two subjects were discarded because of excessive movement. Data were aligned across sessions by coregistering the whole-brain anatomical MR image acquired on each session with the high-resolution anatomical MR image of each subject's brain with custom software (Nestares and Heeger 2000). Cortical surface models of each individual subject's brain (used for visualization and visual area identification) were reconstructed from the high-resolution anatomical MR images with the public domain software SurfRelax (Larsson 2001).

### fMRI Data Analysis

Data from the adaptation scans were analyzed separately for individual subjects and visual area regions of interest (ROIs) (see *Identification of Visual Area ROIs* below) with custom software written in MATLAB. First, the average response time courses to the adapter and probe stimuli for each of the nine trial types (SAME, DIFF × 4 offset conditions + BLANK) were estimated by linear deconvolution (Burock and Dale 2000). Second, the response amplitudes to individual probe stimuli were estimated by a general linear model (GLM). These responses were used to test for significant effects of stimulus condition on response adaptation. To quantify the spatial extent of stimulus-selective response adaptation, we fit a model of neuronal population responses to the average responses for each stimulus type and spatial offset (see appendix).

### Response Time Courses and Amplitudes

The methods used to estimate response time courses and amplitudes were similar to those of a previous fMRI adaptation study (Larsson and Smith 2012); therefore only a brief description is provided here. Average stimulus-evoked response time courses to each of the nine trial types (SAME, DIFF × 4 offset conditions + BLANK) were computed for each subject, ROI, and stimulus condition (NM, BM, SO) separately, with linear deconvolution (Burock and Dale 2000). For each ROI, the mean fMRI response time course was fit with a linear model using least-squares regression to yield an estimate of the mean fMRI response amplitude at each of the 16 time points (0–22.5 s) following trial onset for each composite trial type (i.e., adapter + probe or blank) (Fig. 1*C*).

The average hemodynamic response components corresponding to the probe and to the adapter stimuli were computed from the average time courses as follows. For each subject and ROI, the estimated response time course for the BLANK trials, corresponding to the average response to the adapter stimulus alone, was subtracted from the mean time courses of the eight other trial types, yielding for each trial type the mean response to the probe stimulus alone (Fig. 1*C*). The adapter time courses and the probe time courses were then averaged over ROIs and stimulus conditions (hence weighted by response amplitude in both cases) and were each fit with a synthetic hemodynamic response function (HRF) (a difference of two gammas), convolved with a boxcar function representing the duration of the adapter and probe stimuli. These “average HRFs,” which were derived separately for each subject, were then used to estimate the relative response amplitudes to the different probe stimuli for each individual trial, as described below.

The average adapter response across all trial types was modeled by as a single covariate (column in the design matrix), created by convolving the average HRF for the adapter response with a vector with 1 at the onset time of each trial and 0 elsewhere, and the responses to individual probe stimuli were modeled by separate covariates (columns), created by convolving the average HRF for the probe stimuli with a vector having a value of 1 at the onset of the probe stimulus for a single trial and 0 elsewhere. Estimating probe stimulus responses for individual trials permitted the use of bootstrapping procedures to compute standard errors and confidence intervals of probe responses and model fits. The response time course was then fit with this model by least-squares regression to yield a vector of beta weights, in which the weights corresponding to probe stimuli represented the response amplitudes for individual probe stimulus presentations. Beta weights were averaged across all instances of each probe type for each subject, to yield a mean response amplitude for all eight probes (SAME/DIFF × 4 offset conditions), for all ROIs, and for all three stimulus conditions. We assessed the spatial extent of adaptation by comparison of these mean beta values, averaged across subjects. To determine statistical significance of task-induced changes in response amplitudes, the beta weights for each stimulus condition (averaged across all repeats within each subject) were submitted to a two-way repeated-measures analysis of variance (ANOVA) with probe type (SAME/DIFF) and adapter-probe offset as factors. Significance tests of pairwise differences between conditions (post hoc *t*-tests) were computed using the response amplitudes averaged across repeats within each scan for each subject (10 measurements/subject). Correction for multiple comparisons was done by false discovery rate (FDR) correction methods (Benjamini and Hochberg 1995). To compute interareal correlations in responses or adaptation amplitudes, we used the scan-averaged response amplitudes for SAME or DIFF trials (or the pairwise difference between DIFF and SAME trials for each scan as a measure of adaptation amplitudes). For these analyses data were pooled across subjects, as there were no systematic intersubject differences in response amplitudes or adaptation amplitudes; however, we also carried out these analyses in individual subjects to confirm that the effects were evident also in single-subject analyses.

### Identification of Visual Area ROIs

Standard phase-encoded retinotopic mapping methods were used to identify borders between retinotopic visual areas corresponding to reversals in visual field maps (Larsson and Heeger 2006; Sereno et al. 1995). High-contrast radial checkerboard patterns were presented within 22.5° rotating-wedge apertures or within expanding or contracting 1°-wide rings. For each subject a minimum of three runs each of the rotating wedge stimuli (half clockwise and half counterclockwise) and ring stimuli (half expanding and half contracting) were acquired. Data were preprocessed in the same way as for the adaptation data. Maps of phase (corresponding to polar angle and eccentricity) and coherence (correlation with best-fitting sinusoid) were visualized on computationally flattened representations of the occipital cortical surfaces (“flat maps”) of each individual subject. Boundaries between retinotopic visual areas were drawn by hand on these flat maps following the conventions of Larsson and Heeger (2006) and Wandell et al. (2007). ROIs were combined across left and right hemispheres.

At the start of each adaptation scanning session, a localizer scan was run to identify voxels in each ROI that were responsive to each stimulus type. Stimuli consisted of arrays of patches of the same type as the adapter stimuli. A concurrent RSVP task (same as for adaptation scans) was used to control for attentional level and gaze. The scan commenced with a blank screen for 12 s, followed by five cycles of stimulus presentation (12 s) alternating with a blank screen (12 s). Patches were randomly permuted between locations and random orientations every 1.5 s. Localizer scan data were preprocessed as for the adaptation data and analyzed separately for each session by fitting individual voxel time series with a sinusoid at the stimulus frequency. This yielded for each voxel an estimate of the response amplitude, response phase, and coherence. Visual area ROIs defined by retinotopic mapping were restricted to include only voxels with a coherence ≥0.25 in the localizer scans.

To delineate MT, a separate MT localizer experiment was also run in all subjects. The MT localizer used a block design comprising 10 cycles of “coherent-motion” and “no-motion” epochs, lasting 12 s each. The stimulus consisted of 500 dots (half black and half white, each subtending ∼0.1°) displayed inside an annular aperture (inner radius 1°, outer radius 10°) on a uniform gray background. A 1°-wide fixation cross was shown in black at the center of the stimulus. During coherent-motion epochs, dots moved radially inward then outward at a speed of 1°/s, changing direction every 1.5 s. Dots had a lifetime of 160 ms. During no-motion epochs, stationary dots randomly appeared within the circular region, with the same lifetime as during the motion epochs. No task was used during presentation of this stimulus. Data were analyzed as for the localizer experiment described above. The MT ROI was drawn by hand on each subject's flat map on the basis of the MT localizer scan and the retinotopic localizer scans. The MT ROI was restricted for each stimulus condition according to session-specific localizer activity, in the same way as the other ROIs.

### Modeling Spatial Extent of Adaptation

To quantify the spatial specificity of adaptation for each stimulus type, we fit the average fMRI responses for the SAME and DIFF conditions with a simplified model of the neuronal population response. We used the model to derive estimates of the spatial specificity of adaptation for each visual area, expressed as the average spatial extent of the visual field where an adapter stimulus induces a change (reduction or facilitation) in the response to probe stimuli. Extending the terminology of Priebe et al. (2002), we refer to this spatial extent as the population adaptation field, or pAF. Analogous to population RFs (pRFs), which are defined as the sum of the individual RFs of a population of neurons (Dumoulin and Wandell 2008; Larsson and Heeger 2006), pAFs represent the sum of the individual adaptation fields (Priebe et al. 2002) of those neurons. Unlike pRFs, which reflect the response properties of the area being measured, pAFs are related to the pRFs of the neuronal population where adaptation originated rather than those of the neuronal population under measurement. If adaptation is intrinsic to (originates in) the area being measured, the spatial extent of pAFs will reflect the extent of pRFs in that area. However, if adaptation is inherited from an earlier area with smaller pRFs, pAFs will be smaller than pRFs in the area being measured and instead match the size of pRFs in the earlier area. A comparison of the relative widths of pAFs thus provides a measure of where adaptation originates. By using our adaptation data to derive the spatial extent of the adapting population, we were able to examine whether the adaptation measured in different cortical areas was likely to have been inherited from a single area (i.e., matching pAF sizes across areas) or to be intrinsic to the area from which the data were measured (i.e., pAF sizes varying across areas in agreement with known variance in pRF size). Specifically, we predicted that if adaptation in extrastriate areas originated in V1, the size of extrastriate pAFs should match V1 pAFs; conversely, if some or all of adaptation was intrinsic to extrastriate cortex, extrastriate pAFs should be larger than those of V1, reflecting the larger pRFs in extrastriate visual areas. Details of the model and model fitting procedures are given in the appendix.

## RESULTS

**Fig. 2.**
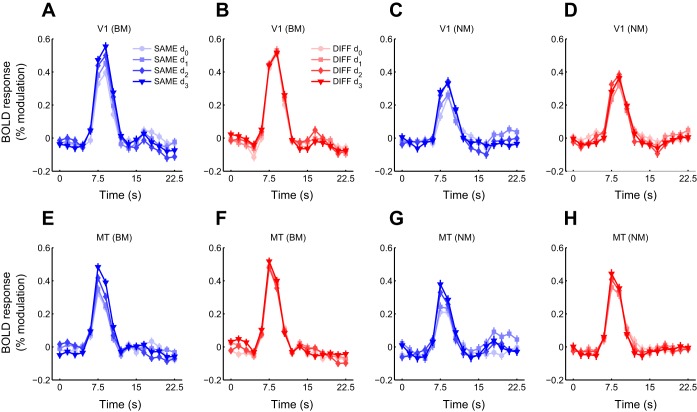
Adaptation to BM and NM in V1 and MT is direction selective and spatially specific. *A*: time courses of responses (averaged across subjects) to BM probe stimuli in V1 having the same direction as the adapter stimulus (SAME trials). Responses to probe stimuli were estimated by subtracting the average response time courses on BLANK (adapter only) trials (Fig. 1*C*) from the average trial-triggered response time courses. Peak response magnitudes increase monotonically with increasing offset between adapter and probe. Error bars, standard error of the estimate (average across subjects). *B*: same as *A* but for probe stimuli having the opposite direction to the adapter stimulus (DIFF trials). Peak response magnitudes do not vary significantly between different adapter-probe offsets. *C*: same as *A* but for NM probes. Peak response magnitudes increase with increasing offset between adapter and probe, but the effect is less pronounced than for BM stimuli. *D*: same as *B* but for NM stimuli. Peak response magnitudes for DIFF trials increase with increasing offset between adapter and probe. *E–H*: same as *A–D* but for area MT. Responses in MT follow the same pattern as those of V1.

**Fig. 3.**
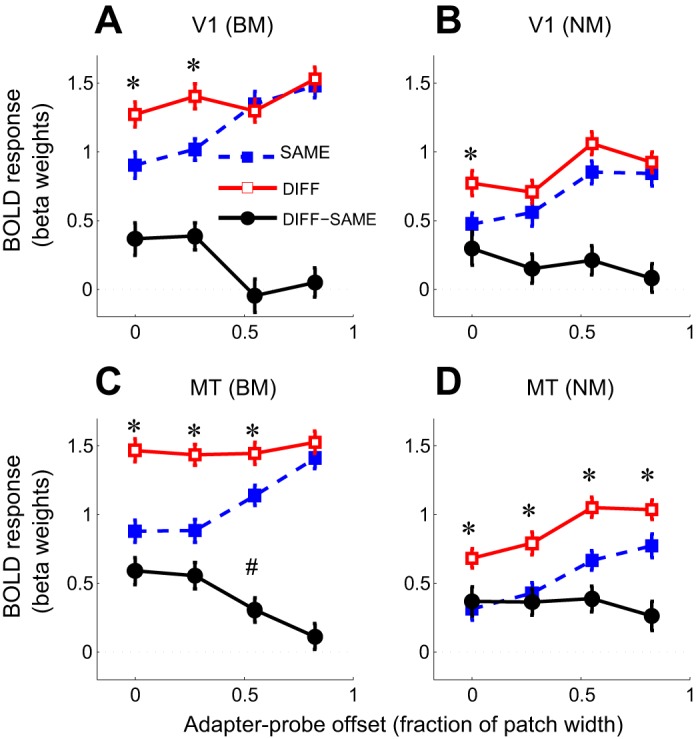
Spatial specificity of direction-selective adaptation to BM and NM in V1 and MT. *A*: mean amplitudes of responses [beta weights of general linear model (GLM) fit] to individual BM probe stimuli in V1 as a function of stimulus direction (SAME or DIFF) and adapter-probe offset. Error bars, SE across trials. Responses to both SAME trials and DIFF trials increase with increasing adapter-probe offset. Black circles, magnitude of direction-selective adaptation (DIFF − SAME) as function of adapter-probe offset (spatial adaptation profile). *Significantly greater response to DIFF than SAME trials (paired *t*-test, corrected for false discovery rate at *P* < 0.05). Note that direction-selective adaptation is spatially specific (only evident at small offsets). *B*: same as *A* but for BM. *C*: same as *A* but for area MT. Direction-selective adaptation in MT is less spatially specific (more broadly spatially tuned) than in V1 (adaptation greater than 0 at all except the largest offset). ^#^Significantly greater adaptation amplitude in MT than in V1 (paired *t*-test, corrected for false discovery rate at *P* < 0.05). *D*: same as *B* for MT. Direction-selective adaptation to NM is evident at all adapter-probe offsets.

Data of 2 of the 11 subjects were discarded because of excessive head motion (greater than ∼5 mm; estimated from the motion parameters output by the motion correction algorithm). The following results are therefore based only on the remaining nine subjects. Both BM and NM stimuli evoked robust fMRI responses in V1 and MT (Fig. 1*C* and Fig. 2). These responses varied both with stimulus condition, being larger when probe stimuli had the opposite direction of motion to the adapter stimuli (DIFF trials) than when the direction was the same (SAME trials), and with adapter-probe offset, being smallest when probe stimuli were presented at the same location as the adapter stimuli and largest when adapter and probes were maximally separated (Fig. 2). The effect of adaptation on the magnitude of event-related time courses shown in Fig. 2 was also seen in the mean response amplitudes estimated for individual trials by a GLM fit (see materials and methods) (Fig. 3). We used these mean responses (averaged within each scan) for the statistical analyses reported below.

### Adaptation to Motion in V1 and MT Is Direction Selective and Spatially Specific

Direction-selective adaptation, evident as stronger fMRI responses to motion probe stimuli having the same motion direction as the adapter stimulus (SAME) compared with probe stimuli having the opposite motion direction (DIFF), was observed both in V1 and MT and for both BM and NM stimuli (Figs. 2 and 3). These differences were all statistically significant [repeated-measures ANOVA, main effect of stimulus direction; V1, BM: *F*(1,8) = 6.56, *P* = 0.034; MT, BM: *F*(1,8) = 30.8, *P* < 0.001; V1, NM: *F*(1,8) = 11.4, *P* = 0.0096; MT, NM: *F*(1,8) = 21.3, *P* = 0.0017].

In both V1 and MT, responses to motion stimuli also showed evidence of spatially specific adaptation: responses to parallel probe stimuli (SAME trials) were weakest when the probes were presented in the same spatial locations as the adapter stimuli, becoming increasingly stronger with increasing adapter-probe offset (Figs. 2 and 3). fMRI responses to opposite motion directions (DIFF trials) showed a similar, but weaker, increase with distance for NM in both V1 and MT, but the effect was much less evident for BM, especially in MT, where responses were essentially constant across the four adapter-probe offsets (Fig. 3*C*). Overall, the effect of varying adapter-probe distance on responses was highly statistically significant for both areas and motion stimulus directions [repeated-measures ANOVA, main effect of adapter-probe offset; V1, BM: *F*(3,24) = 5.15, *P* = 0.0069; MT, BM: *F*(3,24) = 6.26, *P* = 0.0027; V1, NM: *F*(3,24) = 5.31, *P* = 0.0060; MT, NM: *F*(3,24) = 9.14, *P* < 0.001]. The difference in spatial specificity of adaptation between SAME and DIFF trials was statistically significant for BM [repeated-measures ANOVA, interaction between stimulus direction and adapter-probe offset; V1: *F*(3,24) = 3.05, *P* = 0.048; MT: *F*(3,24) = 4.73, *P* = 0.0099] but not for NM [V1: *F*(3,24) = 0.78, *P* > 0.5; MT: *F*(3,24) = 0.34, *P* > 0.7]. Post hoc pairwise *t*-tests were consistent with these results, showing that DIFF responses were significantly greater than SAME responses at zero offset for all stimulus conditions (*P* < 0.05, FDR corrected for multiple comparisons) but that this difference became nonsignificant at larger offsets except for in MT, where responses to NM remained significantly stronger for DIFF than SAME directions even at the largest offset (Fig. 3*D*).

The combined spatial specificity and direction selectivity of adaptation that we observed is consistent with fMRI responses that reflect the population responses of V1 and MT neurons tuned for spatial location and/or motion direction. Specifically, the results are consistent with motion-evoked fMRI responses being composed of the sum of a direction-selective and spatially selective component that is maximally attenuated when the direction and position of probe stimuli are the same as those of the adapter and a spatially selective, but not direction-selective, component that is maximally attenuated when adapter and probe stimuli are presented in the same location and increases with increasing adapter-probe distance. We interpret the first component as reflecting responses of direction-selective subpopulations of neurons tuned to the adapting stimulus direction and spatiotemporal contrast. The second component likely reflects responses of neurons not selective for motion direction but responding only to the spatiotemporal contrast of the adapter stimulus patches. Both of these subpopulations are spatially tuned: only those neurons whose RFs overlap with the adapter stimulus patches are driven by, and hence adapt to, that stimulus.

By definition, the non-direction-selective response component would not differ between the two motion direction conditions (SAME or DIFF), allowing the direction-selective component to be isolated by subtracting responses to SAME trials from responses to DIFF trials for each adapter-probe offset. The result of this subtraction (Fig. 3, black curves) shows the magnitude of direction-selective adaptation as a function of adapter-probe distance and provides a measure of the spatial selectivity of the direction-selective component (note that this is equivalent to the combined effects of stimulus direction and the interaction between stimulus direction and adapter-probe offset in the ANOVA analysis above). In the following, we refer to this plot as the spatial adaptation profile. For BM, the spatial adaptation profiles in both V1 and MT showed maximal adaptation when adapter and probe stimuli were shown in the same locations, gradually decreasing with increasing adapter-probe distance to near zero adaptation at the largest offset (Fig. 3). For NM, the magnitude of direction-selective adaptation was largely constant across adapter-probe offsets, although there appeared to be a slight decrease in adaptation from the smallest to the largest adapter-probe offsets similar to that for BM (Fig. 3, *B* and *D*). However, this difference was not statistically significant, as indicated by the nonsignificant test for interaction between stimulus direction and adapter-probe offset reported above. The lack of clear evidence for spatial tuning of direction-selective adaptation for NM could be due to the underlying neuronal responses lacking spatial tuning, although this is unlikely given the well-established retinotopic organization of V1 and MT. A more likely interpretation is that the RFs of these neurons are larger than the range of adapter-probe offsets, resulting in little recovery from adaptation even at the maximum offset distance. It is also possible that the weaker responses to NM, compared with BM, masked spatially specific variations in responses.

### Adaptation to Motion Direction Can Occur at Multiple Levels of Visual Processing

We capitalized on the spatial specificity of direction-selective adaptation to determine whether there was any evidence in our data for intrinsic MT adaptation. If direction-selective adaptation in MT were due solely to inherited V1 adaptation, then the spatial adaptation profile of MT should be a scaled copy of that of V1—specifically, the spatial extent of direction-selective adaptation should reflect the smaller RF sizes in V1 rather than those in MT. Conversely, if additional direction-selective adaptation took place in MT over and above that inherited from V1, then the spatial adaptation profile would be expected to reflect the larger RF sizes in MT, meaning that direction-selective adaptation should extend over a larger adapter-probe distance in MT than in V1. By comparing the spatial adaptation profiles for the two areas we could thus distinguish these two possibilities.

The spatial adaptation profiles for V1 and MT were similar for both types of motion stimuli but differed slightly for the BM stimuli in that adaptation attenuated more slowly with adapter-probe distance in MT than in V1: at the second-largest offset, there was no significant direction-selective adaptation in V1, but adaptation in MT was still nonzero (Fig. 3*C*). We confirmed that this difference was statistically significant by comparing the magnitude of direction-selective adaptation in V1 and MT (normalized to have the same maximum adaptation magnitude) at each adapter-probe offset. At the second-largest offset, direction-selective adaptation in MT was significantly larger than in V1 [paired *t*-test, *t*(88) = 2.61, *P* = 0.0125, FDR corrected (Benjamini and Hochberg 1995)]. There was no significant difference in adaptation magnitude between V1 and MT at other offsets (*P* > 0.5, FDR corrected), or for NM at any offset (*P* > 0.1, FDR corrected). These results are consistent with a proportion of direction-selective adaptation to BM in MT being intrinsic to that area, whereas for NM adaptation is solely inherited from V1, in agreement with single-unit recordings in macaque MT (Kohn and Movshon 2003; Priebe et al. 2002).

### Estimating Spatial Extent of Direction-Selective Adaptation

**Fig. 4.**
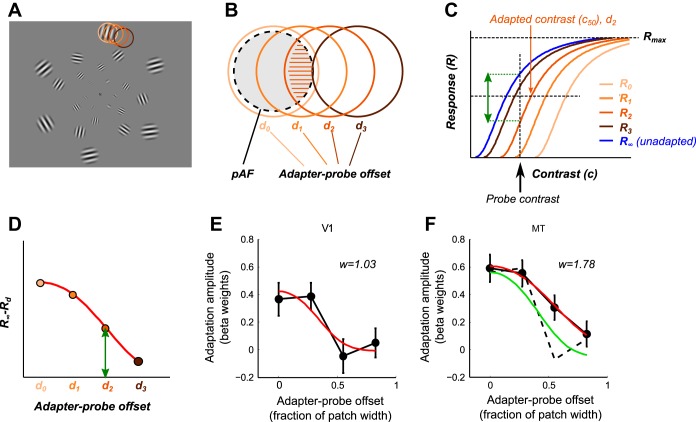
Model-based estimation of population adaptation field sizes in V1 and MT. *A*: example of grating adapter stimulus (NM and SO conditions). Colored circles indicate the 4 different spatial offsets shown for 1 of the stimulus patches (darker colors indicate larger offsets). *B*: schematic illustration of relationship between population adaptation field (pAF) size and spatially specific adaptation. The magnitude of adaptation as a function of adapter-probe offset *d* is given by the strength of stimulus drive from the adapter stimulus, computed as the proportional overlap (hatched area) between the adapter stimulus patch (solid orange circles) and the pAF (light gray circle with dashed black outline). *C*: effect of adaptation on evoked responses. Each curve represents the contrast-response function following prolonged exposure to the adapter at 1 of the 4 offsets (orange curves) or with no adaptation (blue curve). In the model, adaptation serves to change the gain of responses by centering the contrast-response curves horizontally on the adapted contrast. *D*: model prediction of adaptation amplitudes. For each offset, the predicted adaptation amplitude corresponds to the difference between the unadapted response (blue curve in *C*) and the adapted response (orange curves in *C*) at the probe stimulus contrast. *E*: model fit to V1 data for BM stimuli. Black circles and lines indicate V1 adaptation amplitudes as a function of adapter-probe offset (spatial adaptation profile). Model prediction is indicated by red curve. *F*: model fit to MT data for the same stimulus. Dotted black line, linear prediction assuming adaptation inherited from V1 (V1 spatial adaptation profile scaled to the maximum MT adaptation amplitude). Green curve, model fit constrained to use same pAF size as V1 but allowing contrast-response function to vary.

The differences in spatial adaptation profiles between V1 and MT can be qualitatively accounted for by differences in the average RF width of the adapting neuronal populations, referred to as the pAF width. It is, however, not possible to directly infer pAF width from the adaptation profiles. To obtain a quantitative estimate of this parameter, we fit a simplified model of neuronal responses to the data from V1 and MT that incorporated the pAF width of direction-selective neurons as a free parameter. In this model (described in full in appendix), the magnitude of direction-selective adaptation is determined by the strength of responses to the adapter stimulus, which in turn is a function of the position and pAF width of the adapting neurons relative to the adapter stimulus patch size. Because the stimulus patch size was scaled with eccentricity and pRF width is roughly proportional to eccentricity (Dumoulin and Wandell 2008), pAF width in the model is expressed as a constant angular width, allowing the model to be fit to data averaged across eccentricities (i.e., across all voxels in each visual area ROI). pAFs were modeled as circular Gaussians analogous to pRF methods (Dumoulin and Wandell 2008; Larsson and Heeger 2006). For BM, this model provided an adequate fit to the spatial adaptation profiles in both V1 and MT (Fig. 4, *E* and *F*), accounting for a significant proportion of variability of adaptation magnitudes both on the group-average data (computed on mean-centered data averaged across sessions within each subject; V1: *R*^2^ = 0.22, *P* = 0.0036; MT: *R*^2^ = 0.37, *P* = 0.0001) and for most individual subjects (V1: median *R*^2^ = 0.45, range 0.01–0.97; MT: median *R*^2^ = 0.51, range 0.12–0.88). In agreement with the interpretation that the larger spatial extent of direction-selective adaptation in MT than V1 reflected adaptation of MT neurons with larger RF sizes, the estimated pAF size for MT (1.78 times the angular patch width) was nearly twice as large as that for V1 (1.03). This difference was statistically significant (bootstrap test, *P* < 0.05). Although this difference in pAF size is much smaller than the difference between single-neuron RF sizes in V1 and MT, this is to be expected given that the spatial adaptation profile for MT reflects a mix of inherited V1 adaptation and adaptation intrinsic to MT. The estimated pAF width for V1 translates to a pRF size [expressed as full width at half-maximum (FWHM) of a Gaussian RF] of 1.03° at the smallest stimulus eccentricity (3°), increasing to 4.30° at the largest eccentricity (12.5°). Although fMRI measurements of pRF size vary substantially between studies (Dumoulin and Wandell 2008; Larsson and Heeger 2006; Zuiderbaan et al. 2012), this estimate is in broad agreement with the reported range of V1 pRF sizes, consistent with the interpretation that the direction-selective adaptation was intrinsic to V1.

The results of the model fit were consistent with differences between V1 and MT spatial adaptation profiles being due to differences in pAF size (and hence to adaptation taking place both in V1 and MT), but we also considered whether differences in the contrast-response functions of V1 and MT could account for the observations. Specifically, it is known that MT has a more rapidly saturating contrast response than V1 (Tootell et al. 1995), which might distort inherited V1 adaptation effects in a way that could mimic a larger pAF size. We explored this possibility by fitting the model to MT data but constraining the pAF width to be the same as V1, while allowing the saturation of the modeled contrast-response function (expressed as the exponent in the underlying function; see appendix) to vary freely. [Note that for the original model fit, we used a fixed exponent of 2, consistent with previous studies (Gardner et al. 2005)]. This model, however, did less well than the original model in that it systematically underestimated the amount of adaptation in MT and predicted a more rapid drop-off in adaptation with adapter-probe distance, failing to account for the nonzero adaptation at the largest offset (Fig. 4*F*). Moreover, as reported below, our results show a proportional relationship between V1 and MT responses for both types of motion stimuli, which is inconsistent with substantial differences in contrast response between the two areas. A difference in contrast response between V1 and MT is therefore unlikely to underlie the observed differences in spatial adaptation profiles.

We also considered the possibility that differences in RF size between V1 and MT might have given rise to the observed difference in spatial specificity of adaptation in V1 and MT even in the absence of intrinsic MT adaptation. Patterson et al. (2014b) reported that small grating stimuli induce relatively stronger adaptation effects than larger gratings in V1 and MT, implying that for a given (small) stimulus size, areas with larger RFs (e.g., MT) would be more susceptible to adaptation than areas with small RFs (e.g., V1). However, while this might explain the larger overall magnitude of direction-selective adaptation in MT than in V1, it could only explain the difference in shape of the spatial adaptation profiles of these two areas if the contrast-response function of the areas also differed (because a proportionally stronger MT response adaptation would simply scale the V1 spatial adaptation profile upward). As we found no evidence of differences in the shape of contrast-response functions between V1 and MT, differences in the susceptibility to adaptation per se cannot account for our results.

Because the spatial adaptation profiles for NM were essentially flat across all adapter-probe offsets, it was not possible to fit the model to these data. However, the relative similarity in shape between V1 and MT spatial adaptation profiles (Fig. 3, *B* and *D*) and the lack of any significant differences in direction-selective adaptation between V1 and MT for any adapter-probe offset suggest that MT adaptation to NM is inherited from V1, with little evidence of additional adaptation intrinsic to MT. An alternative possibility is that pAFs for NM in MT are much wider than the largest spatial offset tested, resulting in the relatively flat spatial adaptation profile for this area (Fig. 3*D*). This interpretation, if correct, would suggest that a proportion of adaptation to NM is intrinsic to MT, just as for BM. In summary, these results are therefore in agreement with the interpretation that for both BM and NM direction-selective adaptation in MT is largely inherited from V1, but that at least for BM there is also evidence of a small proportion of adaptation taking place within MT.

### Adaptation in V1 Propagates in a (Largely) Nonselective Manner to Extrastriate Visual Areas

**Fig. 5.**
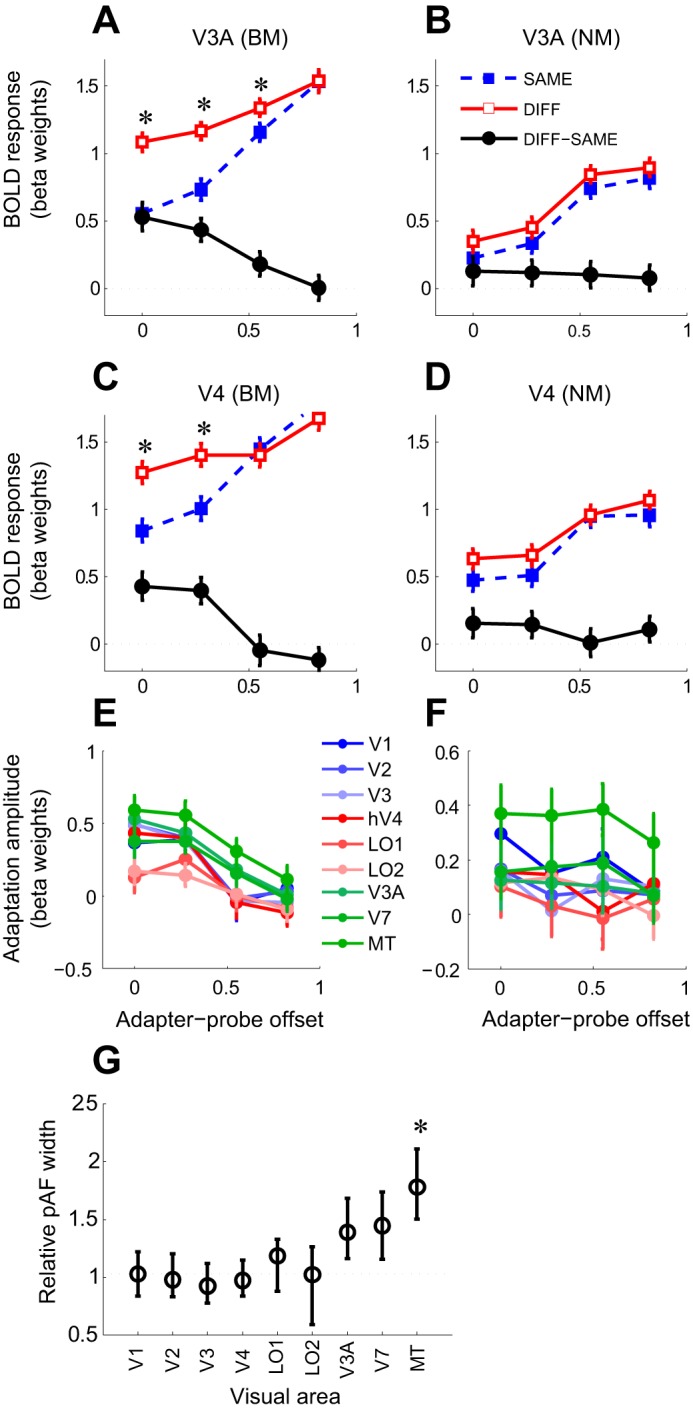
Spatial specificity of direction-selective adaptation in extrastriate visual areas. *A–D*: direction-selective adaptation to BM and NM stimuli in V3A and V4. *Significantly greater response to DIFF than SAME trials (paired *t*-test, corrected for false discovery rate at *P* < 0.05). Symbols as in Fig. 3. *E* and *F*: spatial adaptation profiles across V1 and extrastriate visual areas for BM (*E*) and NM (*F*). *G*: estimated pAF sizes for BM stimuli for V1 and extrastriate visual areas. *Significantly greater extrastriate pAF size than in V1. Except for MT, extrastriate pAF sizes do not differ significantly from V1 pAF estimate, consistent with adaptation being inherited from V1. Only in MT is pAF size significantly greater than in V1, indicating additional direction-selective adaptation intrinsic to area MT.

Our data suggest that direction-selective adaptation in V1 is propagated to MT, profoundly influencing fMRI responses in this area. Given that MT neurons receive strong afferent input from direction-selective V1 neurons, it is perhaps not surprising that adaptation of these afferents should have a pronounced influence on MT responses. However, it is not clear whether the responses of other extrastriate areas having weaker or no selectivity for motion direction should be equally strongly influenced by motion adaptation in V1. We investigated this question by repeating the analysis performed for MT above for seven retinotopic visual areas (V2, V3, V4, LO1, LO2, V3A, and V7) identified in the same subjects. In most extrastriate visual areas, significant direction-selective adaptation was observed both for BM and NM (Table 1; repeated-measures ANOVA, main effect of stimulus direction). The main exception to this pattern was in LO1 and LO2, neither of which showed a significant effect of stimulus direction. Areas V4 and V3A showed a significant effect of stimulus direction for BM but not for NM (Table 1). For BM, there was also evidence of spatially specific direction-selective adaptation in every area except LO1 and LO2 (Table 1; repeated-measures ANOVA, interaction between spatial offset and stimulus direction). Consistent with adaptation originating in V1 for this stimulus, the spatial adaptation profiles of most areas except LO1 and LO2 were similar to a scaled copy of the V1 spatial adaptation profile (Fig. 5, *A* and *E*).

**Table 1.** Results of ANOVA of BOLD response amplitudes by stimulus condition

	V1	V2	V3	V4	LO1	LO2	V3A	V7	MT
BM: direction	***F***_1,8_ **= 6.56**	***F***_1,8_ **= 14.7**	***F***_1,8_ **= 12.9**	***F***_1,8_ **= 11.7**	*F*_1,8_ = 1.86	*F*_1,8_ = 2.42	***F***_1,8_ **= 23.0**	***F***_1,8_ **= 15.9**	***F***_1,8_ **= 30.8**
	***P* = 0.034**	***P* = 0.0050**	***P* = 0.0071**	***P* = 0.0091**	*P* = 0.21	*P* = 0.16	***P* = 0.0014**	***P* = 0.004**	***P* = 0.0005**
BM: offset	***F***_3,24_ **= 5.15**	***F***_3,24_ **= 10.8**	***F***_3,24_ **= 14.1**	***F***_3,24_ **= 20.1**	***F***_3,24_ **= 7.86**	***F***_3,24_ **= 8.10**	***F***_3,24_ **= 23.5**	***F***_3,24_ **= 12.1**	***F***_3,24_ **= 6.26**
	***P* = 0.0069**	***P* = 0.0001**	***P* < 0.0001**	***P* < 0.0001**	***P* = 0.0008**	***P* = 0.0007**	***P* < 0.0001**	***P* < 0.0001**	***P* = 0.0027**
BM: direction × offset	***F***_3,24_ **= 3.05**	***F***_3,24_ **= 3.05**	***F***_3,24_ **= 6.31**	***F***_3,24_ **= 6.54**	*F*_3,24_ = 1.73	*F*_3,24_ = 1.75	***F***_3,24_ **= 5.03**	***F***_3,24_ **= 6.12**	***F***_3,24_ **= 4.73**
	***P* = 0.048**	***P* = 0.0050**	***P* = 0.0026**	***P* = 0.0022**	*P* = 0.19	*P* = 0.18	***P* = 0.0076**	***P* = 0.0031**	***P* = 0.0099**
NM: direction	***F***_1,8_ **= 11.4**	***F***_1,8_ **= 8.86**	***F***_1,8_ **= 7.15**	*F*_1,8_ = 3.33	*F*_1,8_ = 0.82	*F*_1,8_ = 1.88	*F*_1,8_ = 3.76	***F***_1,8_ **= 6.06**	***F***_1,8_ **= 21.3**
	***P* = 0.0096**	***P* = 0.018**	***P* = 0.028**	*P* = 0.11	*P* = 0.39	*P* = 0.21	*P* = 0.089	***P* = 0.039**	***P* = 0.0017**
NM: offset	***F***_3,24_ **= 5.31**	***F***_3,24_ **= 16.9**	***F***_3,24_ **= 22.2**	***F***_3,24_ **= 12.5**	***F***_3,24_ **= 11.4**	***F***_3,24_ **= 6.32**	**F**_3,24_ **= 25.6**	**F**_3,24_ **= 10.9**	**F**_3,24_ **= 9.14**
	***P* = 0.006**	***P* < 0.0001**	***P* < 0.0001**	***P* < 0.0001**	***P* < 0.0001**	***P* = 0.0026**	***P* < 0.0001**	***P* = 0.0001**	***P* = 0.0003**
NM: direction × offset	*F*_3,24_ = 0.78	*F*_3,24_ = 0.27	*F*_3,24_ = 0.36	*F*_3,24_ = 0.42	*F*_3,24_ = 0.14	*F*_3,24_ = 0.39	*F*_3,24_ = 0.07	*F*_3,24_ = 0.25	*F*_3,24_ = 0.34
	*P* = 0.51	*P* = 0.84	*P* = 0.78	*P* = 0.74	*P* = 0.93	*P* = 0.76	*P* = 0.97	*P* = 0.86	*P* = 0.80
SO: orientation	*F*_1,8_ = 3.41	***F***_1,8_ **= 5.70**	*F*_1,8_ = 4.59	*F*_1,8_ = 2.10	*F*_1,8_ = 0.66	*F*_1,8_ = 0.063	*F*_1,8_ = 1.89	*F*_1,8_ = 0.093	*F*_1,8_ = 0.26
	*P* = 0.10	***P* = 0.044**	*P* = 0.065	*P* = 0.19	*P* = 0.44	*P* = 0.81	*P* = 0.21	*P* = 0.77	*P* = 0.62
SO: offset	*F*_3,24_ = 1.77	***F***_3,24_ **= 8.29**	***F***_3,24_ **= 8.75**	***F***_3,24_ **= 7.93**	***F***_3,24_ **= 3.22**	*F*_3,24_ = 2.14	***F***_3,24_ **= 12.7**	***F***_3,24_ **= 7.74**	***F***_3,24_ **= 15.7**
	*P* = 0.18	***P* = 0.0006**	***P* = 0.0004**	***P* = 0.0008**	***P* = 0.041**	*P* = 0.12	***P* < 0.0001**	***P* = 0.0009**	***P* < 0.0001**
SO: orientation × offset	*F*_3,24_ = 2.35	***F***_3,24_ **= 4.12**	***F***_3,24_ **= 4.82**	***F***_3,24_ **= 3.53**	***F***_3,24_ **= 5.31**	***F***_3,24_ **= 5.75**	***F***_3,24_ **= 5.46**	*F*_3,24_ = 0.79	*F*_3,24_ = 2.33
	*P* = 0.098	***P* = 0.017**	***P* = 0.0091**	***P* = 0.030**	***P* = 0.0060**	***P* = 0.0041**	***P* = 0.0052**	*P* = 0.51	*P* = 0.10

BM, broadband motion; NM, narrowband motion; SO, static orientation. Bold type indicates significant effects (*P* < 0.05).

Estimates of pAF widths in each area (obtained by fitting the same model described above to the spatial adaptation profiles for BM for each area) further supported this interpretation, as no significant differences in pAF width (resampling test, *P* > 0.1) were found between V1 and any extrastriate area other than MT. However, a plot of the estimated pAF widths across areas, sorted in ascending order, revealed a trend for larger pAF sizes in dorsal stream areas (V3A, V7, and MT) (Fig. 5*G*). This could reflect additional adaptation intrinsic to V3A and V7, but it is also possible that these areas receive input from MT that could potentially account for the trend toward larger pAF widths in these areas.

### Adaptation Does Not Change BOLD Response Coupling Between V1 and Extrastriate Areas

**Fig. 6.**
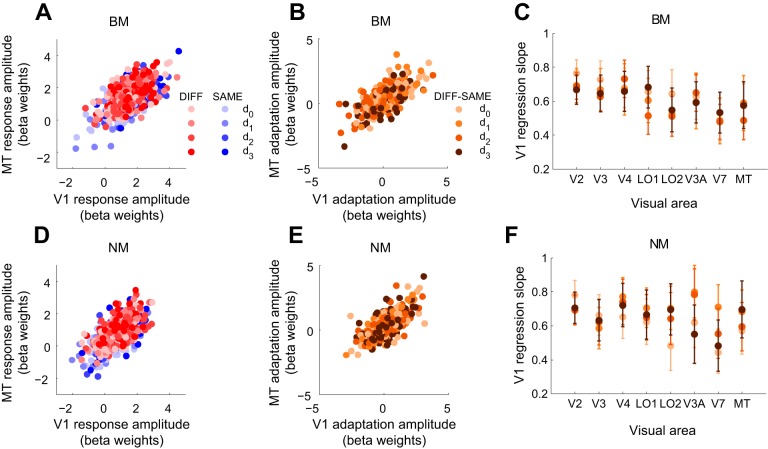
Linear coupling of motion-evoked BOLD responses and direction-selective adaptation between V1 and extrastriate visual areas. *A*: scatterplot of response amplitudes (beta weights of GLM fit) to BM probe stimuli in V1 and MT. Color saturation indicates offset condition (darker circles correspond to larger offsets). Each circle represents the average response across trials within 1 scan and subject. For all offset conditions, MT response amplitudes increase proportionally with V1 response amplitudes. *B*: scatterplot of adaptation amplitudes for BM probe stimuli in V1 and MT (computed as difference between responses to DIFF and SAME trials). Adaptation amplitudes in MT are linearly proportional to V1 adaptation amplitudes. *C*: slopes of linear regression between adaptation amplitudes in V1 and extrastriate visual areas for each adapter-probe offset (color indicates offset as in *B*). Error bars, 95% confidence intervals of regression coefficients. In every area examined confidence intervals for all 4 offsets overlap, indicating no significant difference in slopes as a function of adaptation state. *D–F*: same as *A–C* but for NM.

The analysis of spatial adaptation profiles above suggested that adaptation effects in extrastriate areas are dominated by inherited V1 adaptation, even when there was evidence of additional intrinsic extrastriate adaptation (i.e., adaptation to BM in MT). If extrastriate adaptation is inherited from V1, BOLD responses in V1 and extrastriate areas would be expected to be highly correlated. Consistent with this prediction, MT responses were strongly and linearly correlated with V1 responses for both BM and NM (Fig. 6). This was true both for the responses to SAME and DIFF trials (Fig. 6, *A* and *D*) (SAME trials: BM: *r* = 0.57, *P* < 0.0001; NM: *r* = 0.62, *P* < 0.0001; DIFF trials: BM: *r* = 0.57, *P* < 0.0001; NM: *r* = 0.59, *P* < 0.0001) and for the direction-selective adaptation component obtained by the difference between DIFF and SAME trial responses (Fig. 6, *B* and *E*) (BM: *r* = 0.67, *P* < 0.0001; NM: *r* = 0.69, *P* < 0.0001).

Similar results were found for other extrastriate areas; in every area examined the magnitude of direction-selective adaptation was strongly and significantly correlated with that of V1 (Fig. 6, *C* and *F*), with V1 adaptation accounting for a large proportion (between 45% and 76%) of variability in every area. Moreover, if the strength of coupling between V1 and MT responses had changed as a function of adaptation state (which would depend on stimulus condition and adapter-probe distance), we would have expected a difference in regression slopes between the different adapter-probe offsets. We tested this prediction by an analysis of covariance with spatial offset or stimulus condition (SAME/DIFF) as grouping variable; any differences in slope between offsets or conditions would have been evident as a significant interaction between slope and the grouping variable. No significant interactions were found, either for response amplitudes [BM: *F*(3,704) = 0.66, *P* > 0.5; NM: *F*(3,680) = 2.03, *P* > 0.1] or for adaptation amplitudes (i.e., DIFF-SAME) [BM: *F*(3,348) = 0.53, *P* > 0.6; NM: *F*(3,336) = 0.73, *P* > 0.5]. Our data thus demonstrate that the BOLD coupling between V1 and MT does not change significantly as a result of adaptation state. Similarly, regression slopes between V1 and extrastriate areas did not differ significantly between adapted and unadapted conditions (overlapping 95% confidence intervals in Fig. 6, *C* and *F*; analysis of covariance, *P* > 0.1 FDR corrected for multiple comparisons in all areas), indicating that adaptation in V1 does not result in a change in coupling strength between V1 and downstream areas.

### Orientation-Selective Adaptation Is Also Inherited and Can Be Both Suppressive and Facilitatory

**Fig. 7.**
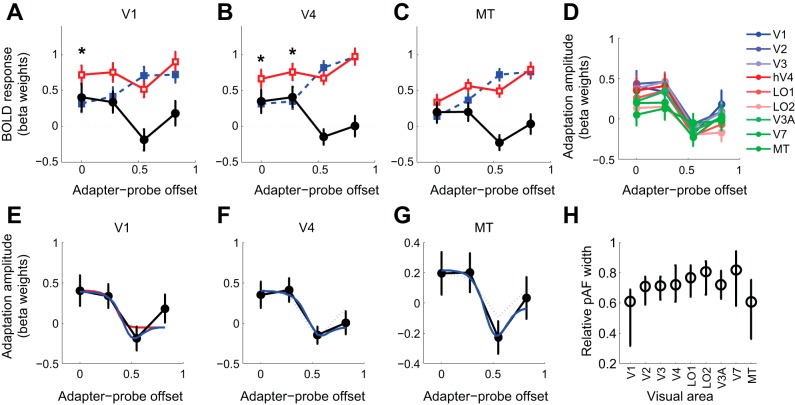
Spatial specificity of orientation-selective adaptation in V1 and extrastriate visual areas. *A–C*: orientation-selective adaptation to grating stimuli in V1, V4, and MT. Symbols as in Fig. 3. *D*: spatial adaptation profiles across V1 and extrastriate visual areas for grating stimuli. *E–G*: model fits to spatial adaptation profiles in *A–C*. *H*: estimated pAF sizes for grating stimuli for V1 and extrastriate visual areas.

Our results demonstrate that direction-selective motion adaptation in V1 is inherited by downstream extrastriate areas regardless of their selectivity for motion. To investigate whether adaptation to other stimulus features is equally broadly propagated, we measured orientation-selective adaptation to grating stimuli identical to those used for the NM condition but varying orientation instead of motion direction and using contrast-reversing gratings instead of drifting gratings. The results of this stimulus manipulation revealed a pattern of adaptation that was overall similar to that for motion but also had some notable differences. First, in all areas, responses to static gratings were weaker and more variable than responses to drifting gratings or moving dots. Second, in V1 there was no significant effect of stimulus orientation or adapter-probe distance on responses (Table 1; repeated-measures ANOVA, *P* > 0.1), although there was a nonsignificant trend toward lower responses for stimuli having the same orientation and position as the adapter (Fig. 7*A*) [Table 1; repeated-measures ANOVA, interaction between stimulus orientation and position, *F*(3,24) = 2.35, *P* = 0.098] [note, however, that a post hoc *t*-test found significantly (*P* < 0.05, FDR corrected) greater activity for DIFF than SAME trials for the zero-offset condition]. The lack of a statistically significant effect in V1 may simply reflect the lower response amplitudes and higher noise in this area, as significant effects of adapter-probe distance and/or the interaction between stimulus orientation and distance were found in most extrastriate areas (Fig. 7, *B* and *C*, Table 1).

Notably, the spatial adaptation profiles in all extrastriate areas were very similar (essentially scaled copies) of that in V1, consistent with adaptation originating in this area (Fig. 7*D*). The spatial adaptation profiles for orientation showed the same overall pattern as those for BM, with adaptation being maximal when probe and adapter stimuli were shown in the same position and becoming weaker as adapter-probe distance increased (Fig. 7*E*). Unlike the results for BM, however, for all areas except V7 and LO2 the smallest adaptation was found not at the maximum offset but at an intermediate (second largest) adapter-probe distance (Fig. 7, *A–D*). Moreover, the adaptation amplitude at this offset was negative, indicating response facilitation [i.e., stronger response to adapted (SAME) than to nonadapted (DIFF) orientation]. Facilitation due to adaptation has been shown to occur as a result of adaptation of suppressive surrounds (Wissig and Kohn 2012), and our results are consistent with such an interpretation. Although our data do not speak to the underlying nature of the surround suppression, the observed spatial adaptation profiles were reasonably well fit by modifying the above-described model to include a subtractive suppressive surround [implemented by replacing the Gaussian pAF with a difference of Gaussians (DoG); see appendix for details] (Fig. 7, *E–G*). Fitting this model to each area yielded estimates of pAF widths that were very similar across visual areas, consistent with adaptation originating in V1, with no evidence of additional adaptation in extrastriate areas (Fig. 7*H*). The same results were obtained with the original model (not including surround suppression), which yielded similar pAF widths across areas, with no evidence for significantly larger pAFs in extrastriate visual areas (data not shown). As for direction-selective adaptation, the magnitude of orientation-selective adaptation in extrastriate areas was linearly proportional to that in V1, and the coupling did not vary with adapter-probe distance (data not shown). In summary, we found evidence that orientation-selective adaptation, like direction-selective adaptation, is propagated from V1 to all extrastriate areas to a similar degree and that both suppression and facilitation due to V1 adaptation are inherited by downstream extrastriate areas.

## DISCUSSION

The results of this study suggest that adaptation-induced changes in human V1 responses are propagated faithfully to downstream extrastriate visual areas, causing profound changes in responses in these areas that mirror those in V1. In most extrastriate visual areas, the spatial specificity of orientation- and direction-selective adaptation did not differ from that in V1, suggesting that adaptation was inherited from V1. In MT, however, the spatial specificity of direction-selective adaptation was broader than in V1, consistent with a combination of inherited V1 adaptation and intrinsic MT adaptation.

### Does Adaptation to Single-Stimulus Features Occur at Multiple Stages in the Visual Processing Stream?

Recording from neurons in macaque MT, Priebe et al. (2002) found that adaptation of transient responses to BM transfers across MT RFs, suggesting adaptation intrinsic to MT. No such transfer of adaptation was observed for sustained responses, which extended only over a distance comparable to V1 RFs, consistent with adaptation inherited from V1. Similarly, Kohn and Movshon (2003) found that adaptation of sustained responses to narrowband gratings in MT could be explained by adaptation of V1 afferents.

Our results are consistent with these earlier studies, suggesting a difference in adaptation mechanisms for BM and NM. Our results also imply that intrinsic adaptation to BM in MT occurs both for short (Priebe et al. 2002) and long (our study) adapter durations. However, the proportion of adaptation not accounted for by V1 was small, as evidenced by the modest difference in spatial specificity of adaptation between MT and V1, indicating that the bulk of adaptation occurs in V1. The small size of the effect may explain why an fMRI study comparing the tuning of adaptation in V1 and MT (Lee and Lee 2012) failed to find conclusive evidence for direction-selective adaptation intrinsic to MT. It is not obvious why adaptation mechanisms should differ between BM and NM, but the fact that we found evidence of intrinsic adaptation in both V1 and MT for one of these motion types indicates that adaptation can occur at multiple processing stages, at least for some stimuli. These results are also consistent with psychophysical evidence for multiple stages of motion adaptation, evidenced by qualitative differences in motion aftereffects when adapting and test stimuli are presented in the same or different locations (Lee and Lu 2012).

However, it is apparent that adaptation at higher levels is modest relative to earlier levels, even in areas that respond strongly to the adapted features. For example, in human visual cortex, area V3A shows a robust motion-evoked BOLD response that is similar in magnitude to that in MT (Larsson and Heeger 2006; Tootell et al. 1997), yet we found at best limited evidence of direction-selective adaptation intrinsic to this area. For orientation, we found no evidence for adaptation beyond V1. These results indicate that adaptation primarily takes place at the first processing stage selective for the feature, with little additional adaptation at subsequent processing levels. It should be emphasized, however, that the ability of our method to detect small differences in spatial specificity of adaptation indicative of multistage adaptation is limited by the relatively low signal-to-noise ratio (SNR) of fMRI BOLD adaptation effects. Hence we cannot rule out that additional adaptation to motion and orientation might occur in extrastriate areas other than MT, but the magnitude of these effects must be small relative to those of V1.

### Inherited V1 Adaptation Can Profoundly Modify Response Properties of Extrastriate Visual Areas

We found that adaptation to orientation or motion direction in V1 can induce orientation- or direction-selective changes in the responses of extrastriate visual areas, including those not strongly selective for either stimulus feature (e.g., V4). This result extends earlier findings (Tolias et al. 2005) that adaptation to motion can induce direction-selective responses in V4 neurons that normally lack such selectivity. Together these results suggest that inherited adaptation can profoundly modify response properties of downstream areas, corroborating psychophysical evidence that inherited low-level adaptation effects can affect higher-level visual processing (Dickinson and Badcock 2013; Xu et al. 2008). If similarly large adaptation effects occur under natural viewing conditions, it would imply that neuronal response properties could potentially change profoundly even during normal vision. A corollary of this finding is that higher neural processing stages are unable to compensate for adaptation-induced changes in afferent input, in that such changes do not result in changes in the way these areas integrate their inputs, as previously shown for V1 spiking responses driven by LGN afferents (Dhruv and Carandini 2014). Our results demonstrate that this holds also for cortico-cortical coupling measured with BOLD fMRI, which showed a strong linear correlation between V1 and extrastriate areas that did not differ between adapted and nonadapted stimulus conditions. This also implies that to the extent that adaptation can induce changes in neurovascular coupling (Moradi and Buxton 2013), such changes are either small or do not significantly influence the strength of interareal coupling.

### Implications for Measuring Stimulus Selectivity with fMRI Adaptation

The experimental design used in this study has similarities to a previous fMRI study by Murray et al. (2006), who failed to find evidence of orientation-selective adaptation in V1–V3 that could not be explained by simple spatially selective adaptation. On the basis of this result they suggested that fMRI adaptation may simply measure vascular adaptation rather than reflecting underlying neuronal adaptation. Our results are inconsistent with this interpretation. Unlike Murray et al. (2006), we found that adaptation induced changes in fMRI responses that were selective both for spatial location and orientation or motion direction, consistent with neural adaptation but not with vascular adaptation. However, we also found that stimulus-selective adaptation effects in V1 are propagated downstream to almost all extrastriate visual areas. Because these effects reflect properties of V1, the magnitude of stimulus-selective adaptation in extrastriate areas does not necessarily reflect intrinsic stimulus selectivity. These results provide a ready explanation for the observation that adaptation to visual shapes in macaque inferotemporal (IT) cortex is much more sharply tuned than neuronal responses to the same stimuli (Sawamura et al. 2006). If adaptation in IT neurons is dominated by inherited V1 adaptation to low-level features, the selectivity of adaptation will primarily reflect the response properties of V1 rather than those of IT. A corollary (and testable prediction) of this explanation is that intrinsic IT adaptation should more closely reflect response selectivity in this area.

The above results imply that any meaningful interpretation of fMRI adaptation data hinges on the ability to distinguish adaptation effects that are intrinsic from those that are inherited. In this study we have demonstrated a method to dissociate inherited from intrinsic adaptation by quantifying the spatial specificity of adaptation. Our method is generally applicable to studying adaptation effects in any sensory modality with RFs varying in size across areas. A different but conceptually related strategy was used by Lee and Lee (2012), who measured tuning of adaptation across multiple visual areas to dissociate inherited from intrinsic adaptation.

A common strategy for minimizing (as opposed to quantifying) the effects of inherited adaptation is to present adapter and probe stimuli at different sizes or locations (e.g., Larsson and Smith 2012; Summerfield et al. 2008). Such differences are typically small enough that many of the contours or features of the adapter and probe stimuli overlap. Because we found that orientation-selective adaptation effects in V1 can be both suppressive and facilitatory (see also Wissig and Kohn 2012), this predicts complex and potentially profound adaptation effects in V1 that would propagate to extrastriate areas. Only very large displacements—e.g., across hemifields—would be expected to be relatively unaffected by inherited adaptation, which may explain in part reported differences in the effects of face and object adaptation within and across hemifields (Kovács et al. 2005). Interestingly, evidence suggests that position invariance of face adaptation depends on the timescale of adaptation (Kovács et al. 2007), raising the question of whether the spatial specificity of adaptation to low-level features such as motion and orientation also varies with adaptation duration.

Although our findings may have particular relevance for the interpretation of fMRI adaptation, the problem of dissociating inherited from intrinsic response properties is inherent in any measurements of neuronal responses. Indeed, evidence suggests that even higher visual areas inherit many low-level response properties of early visual areas, such as sensitivity to position, size, and contrast (DiCarlo and Maunsell 2003; Kravitz et al. 2010; Yue et al. 2011). The combined effects of inherited response properties and inherited adaptation imply that the response properties of higher-level neurons need not be static and fixed but may change dynamically as a result of changes in spatial and temporal context that modulate responses at earlier processing stages.

## GRANTS

This work was supported by Wellcome Trust project grant WT090749MA to J. Larsson.

## DISCLOSURES

No conflicts of interest, financial or otherwise, are declared by the author(s).

## AUTHOR CONTRIBUTIONS

Author contributions: J.L. conception and design of research; J.L. and S.J.H. analyzed data; J.L. interpreted results of experiments; J.L. prepared figures; J.L. drafted manuscript; J.L. and S.J.H. edited and revised manuscript; J.L. and S.J.H. approved final version of manuscript; S.J.H. performed experiments.

## Appendix: MODEL-BASED ESTIMATION OF POPULATION ADAPTATION FIELD SIZE

Population adaptation field (pAF) size was estimated by using a simplified model of neuronal population responses to predict the relative magnitude of stimulus-specific response adaptation at different adapter-probe stimulus offsets (i.e., the spatial adaptation profile) as a function of pAF size. The aim of the model was not to provide a biologically realistic description of the underlying neuronal responses but to test whether differences in pAF size could account for the observed spatial adaptation profiles and whether the resulting pAF sizes were consistent with measurements of pRF sizes in visual cortex.

The basic model had only two free parameters—the maximum average stimulus-evoked response magnitude and the spatial extent of the RFs of the adapting population (i.e., pAF size). We modeled pAFs as circular Gaussians in the first instance; a second model including a suppressive surround was also used (a DoG) that had two additional parameters: spatial extent of surround and relative weight of surround compared with center.

In the model, pAF size (FWHM of circular Gaussian) was expressed as a fraction of stimulus patch size. As adapter-probe displacements were a constant proportion of patch size at all eccentricities, and patch size varied proportionally with eccentricity (meaning they subtended a constant angular width at all eccentricities), by making the assumption that pAF sizes are also proportional to eccentricity we could fit the same model to data averaged across all eccentricities (all voxels) within each ROI (thereby improving the robustness of the fits). (Note that we also assume pAF size to be constant across polar angles, allowing for averaging of data within each eccentricity.) We justified this assumption on the basis of two observations. First, pRF widths (and hence pAF widths) are known to increase linearly with eccentricity and, at higher eccentricities, roughly proportionally to eccentricity (Dumoulin and Wandell 2008; Smith et al. 2001). Second, behavioral measurements of spatially specific adaptation (Ejima and Takahashi 1984, 1985) have found that the sensitivity of grating adaptation to adapter-probe displacements is proportional to the number of grating cycles, rather than the absolute extent of the stimuli, consistent with spatial specificity scaling proportionally with eccentricity. In our stimuli, the number of grating cycles per patch was constant across eccentricities and the spatial offsets were likewise proportional to the number of cycles in each patch, suggesting that the magnitude of sensory adaptation in our data should be constant across eccentricities. We explicitly tested this assumption by measuring spatial adaptation profiles separately for each of the four stimulus eccentricities used (averaging data across voxels within an eccentricity range corresponding to the inner and outer edges of each stimulus patch). If the assumption of proportionality between eccentricity and pAF size were correct, spatial adaptation profiles should be identical across the four stimulus eccentricities. Consistent with this prediction, although data analyzed by eccentricity band were noisier than those averaged across all eccentricities, there were no significant differences between the spatial adaptation profiles across eccentricities in any area for any of the three stimulus types (2-way repeated-measures ANOVA, main effect of eccentricity: *P* > 0.1, interaction between eccentricity and spatial displacement: *P* > 0.1). However, our approach means that the derived estimates of pAF size can only be approximately compared with previous estimates of pRF size by assuming a proportionality between eccentricity and pAF size.

The model assumed that the stimulus-evoked population (fMRI) response could be described as the sum of two global (spatially untuned) and two spatially tuned components:

(1) $$R=R_{0}+R_{\text{g}}+R_{\text{s}}+R_{\text{ns}}$$

The first component, *R*_0_, represents baseline activity. The second component, *R*_g_, is a stimulus-evoked but global (spatially unspecific) response that is assumed to be independent of stimulus contrast or orientation or direction (Donner et al. 2008). The model assumes that the first two components are constant (because they either do not adapt or adapt in a way that is identical for all stimulus conditions). The last two components are spatially tuned and contrast sensitive. The first of these spatially tuned components, *R*_s_, represents the aggregate stimulus-evoked response of neurons responding selectively to the stimulus orientation (SO) or direction (NM/BM) of the stimulus; we refer to this as the stimulus-selective response component. The second spatially tuned component, *R*_ns_, represents the aggregate response of all other visually responsive neurons, whether they be tuned to different stimulus parameters, very broadly tuned, or completely untuned.

We are interested specifically in adaptation of the stimulus-selective component, *R*_s_, as the spatial specificity of adaptation will give us an indirect measure of the extent of the pAF of this subpopulation of neurons. We cannot, using fMRI, observe *R*_s_ directly. However, if we formulate *Eq. 1* separately for our SAME and DIFF conditions (*Eqs. 2* and *3* below), it can be shown that by subtracting one from the other we obtain an expression (*Eq. 4*) that is composed of two measures of stimulus-selective response: the unadapted probe response (which depends only on pAF size and does not vary with spatial offset) and the adapted probe response (which depends both on pAF size and spatial offset).

(2) $$R_{\text{SAME}}\;\left(d,w\right)=R_{0}+R_{\text{g}}+R_{\text{s}}\;\left(d,w\right)+R_{\text{ns}}\;\left(d,w\right)$$

(3) $$R_{\text{DIFF}}\;\left(d,w\right)=R_{0}+R_{\text{g}}+R_{\text{s}}\;\left(\infty ,w\right)+R_{\text{ns}}\;\left(d,w\right)$$

(4) $$R_{\text{DIFF}}\;\left(d,w\right)-R_{\text{SAME}}\;\left(d,w\right)=R_{\text{s}}\;\left(\infty ,w\right)-R_{\text{s}}\;(d,w)$$

Note that the global terms *R*_0_ and *R*_g_ and the nonselective component *R*_ns_ drop out of this rearrangement. Because stimulus orientation (SO) or direction (NM/BM) varied randomly across patches, the average distribution of these stimulus parameters did not differ between SAME and DIFF conditions, and hence the nonselective component *R*_ns_ was identical for both conditions. The left-hand terms in *Eq. 4* correspond to the spatial adaptation profile for each stimulus type.

In the above, the spatially tuned responses have been written as functions of pAF width *w* and adapter-probe distance *d* to make explicit the effects of spatially specific adaptation on the stimulus-evoked response. For DIFF trials, *R*_s_ denotes the response of a population that is selective for the orientation/motion direction of the DIFF probe. As DIFF probes, by definition, have orthogonal stimulus parameters to the adapter, this population does not experience stimulus-selective adaptation. To indicate this, the adapter-probe distance is denoted by ∞, as the corresponding adapter stimulus for this population is never seen, i.e., is at an infinite distance from the probe stimulus.

We can derive predictions for the two stimulus-selective components on the right-hand side of *Eq. 4* so as to arrive at an estimate of the extent of the underlying adapted population, *w*, as described in the following. The stimulus-selective response component *R*_s_ was modeled by a standard Naka-Rushton contrast-response function:

(5) $$R_{s}=R_{\text{0,s}}+R_{\max,\text{s}}\;c^{n}/\left(c^{n}+c_{50}^{n}\right)$$

where *c* is stimulus contrast (ranging from 0 to 1), *R*_0_ is baseline activity, *R*_max_ is maximum activity, *n* is an exponent that describes the slope of the contrast-response function, and *c*_50_ is the contrast at half-maximum response. In this equation adaptation was modeled as changes in contrast gain by varying *c*_50_, holding other parameters constant. Our model makes the assumption that the effect of adaptation is to center the contrast-response function on the average adapter contrast within the pAF. Such a shift is consistent with both single-unit data (e.g., Ohzawa et al. 1982) and fMRI data (Gardner et al. 2005). Although adaptation in single cortical neurons can induce changes both in the maximum response *R*_max_ and the slope *n*, contrast adaptation of the human BOLD fMRI response in early visual areas is well described by a change in *c*_50_ only, with *n* fixed at 2 (Gardner et al. 2005). We adopted this strategy to avoid overfitting; however, fits done with different values of *n* gave qualitatively similar results.

In the model, adaptation is implemented by setting *c*_50_ equal to the effective adapting contrast, which is computed as the inner product (corresponding to the relative overlap, for each offset condition) between the adapter stimulus and the pAF, multiplied by the mean contrast *c*_adapter_ of the adapter stimulus *S* (Fig. 5, *A–C*):

(6) $$c_{50}=c_{\text{adapter}}\;<\text{pAF},\;S>$$

When the distance between the adapter and probe centers increases, the overlap between the adapter stimulus and a pAF that is centered on the probe decreases as a combined function of adapter-probe distance and the spatial extent, *w*, of the pAF. (Although in the experiment it was the probe stimuli that changed location, in considering the response of the pAF to probe stimuli under different offset conditions the reader may find it helpful to consider the probe stimuli as stationary and the adapter as changing location.) Specifically, when the adapter and probe stimuli are in the same location, the effective adapter contrast is maximal, causing maximal adaptation (increased *c*_50_; rightward shift of contrast-response function); conversely, effective adapter contrast is minimal when the adapter is at the maximum distance from the probe (decreased *c*_50_; leftward shift of contrast-response function). In theory, the contrast-response function for a completely nonoverlapping population should be centered on zero contrast, as that is the average contrast of the (nonexistent) adapter; in practice, unadapted neurons tend to have a *c*_50_ greater than zero (Dhruv et al. 2011; Movshon and Lennie 1979; Ohzawa et al. 1982). In our model, we used an unadapted *c*_50_ = 0.05 but verified that the results were qualitatively similar using *c*_50_ = 0.03. The rate and pattern of fall-off in adaptation with increasing adapter-probe distance will thus be a function of the width of the pAF.

The basic, single-Gaussian, model above could not explain observed undershoots in the stimulus-selective responses for one of the stimulus conditions (SO), and so in this case we also evaluated a model that included a suppressive surround to test whether this could better account for the response profiles. Although evidence suggests that the effect of surround suppression on visual cortical neurons is divisive [i.e., a change in contrast or response gain (Cavanaugh et al. 2002; Wade and Rowland 2010)], we chose to model it as a subtractive effect, mainly because such a model is more stable numerically, which was important given the limited number of spatial offsets sampled and the relatively low SNR of fMRI data. Additionally, since the objective of our model was to derive estimates of the size of pAFs it is pertinent that the choice of divisive or subtractive model does not greatly affect the estimated size of the surround (Sceniak et al. 2001).

The suppressive surround component was stimulus selective and was modeled in the same way as the stimulus-selective center component (i.e., assuming same orientation tuning) but with the constraint that the Gaussian describing the surround RF was larger than that of the center. Adaptation of the surround was modeled identically to that of the center, by setting the semisaturation coefficient *c*_50,surround_ of the surround to the effective adapter contrast of the surround. The response of the surround component, weighted by a scale factor, was then subtracted from that of the center to give an overall selective response:

(7) $$R_{\text{s},\text{surround}}=R_{0,s}+R_{\max,s}c^{n}/\left(c^{n}+c_{50}^{n}\right)-k_{\text{S}}\left[R_{0,s}+R_{\max,s}c^{n}/\left(c^{n}+c_{50,surround}^{n}\right)\right]$$

The surround was modeled as having the same *R*_max_ as the center, but the width of the surround and the relative weight of the surround *k*_s_ were free to vary.

We used nonlinear minimization techniques to fit the model parameters against the spatial adaptation profiles for each stimulus type averaged across subjects, minimizing the sum-squared difference between the data and the predicted model response. The goodness of fit was assessed as the proportion of variability explained by the model, evaluated using individual subjects' data. Fits were considered significant if the amount of variability explained was significantly greater than expected by chance (Fisher's *z*-transformation test, α = 0.05).

Bootstrapping procedures were used to compute confidence intervals of the estimated model parameters and differences between parameters. On each bootstrap iteration, response amplitude data were sampled with replacement (using the same number of samples as in the actual measurements) from each subject and the results averaged across subjects for each ROI and adapter-probe distance in the same way as done for the actual measurements. The model was then fit to the resampled data. One thousand bootstrap iterations were run. Parameter confidence intervals (68% and 95%) were computed from the bootstrapped distributions and used to assess significance of the estimates.
